# Authors' Reply to: Errors in Tracing Coronavirus SARS-CoV-2 Transmission Using a Maximum Likelihood Tree. Comment on “A Snapshot of SARS-CoV-2 Genome Availability up to April 2020 and its Implications: Data Analysis”

**DOI:** 10.2196/24661

**Published:** 2020-11-11

**Authors:** Carla Mavian, Simone Marini, Mattia Prosperi, Marco Salemi

**Affiliations:** 1 Department of Pathology, Immunology, and Laboratory Medicine, College of Medicine Emerging Pathogens Institute University of Florida Gainesville, FL United States; 2 Department of Epidemiology, College of Public Health and Health Professions Emerging Pathogens Institute University of Florida Gainesville, FL United States

**Keywords:** SARS-COV-2, phylogeny, cladogram

Before discussing, in detail, the serious technical issues, and conceptual and theoretical mistakes, in the commentary by Forster and Forster [1], we would like to emphasize the following points. First, very recent work by Morel and colleagues [2] has confirmed and further extended our original observation of a lack of a phylogenetic signal in SARS-CoV-2 sequences from the early phase of the pandemic, which is in line with our main criticism that Forster et al’s work [3] was based on a superficial analysis of biased and noisy sequence data, resulting at best in misleading conclusions. Second, many of the claims in the paper by Forster et al [3] have been criticized by three independent Letters to the Editor published in the *Proceedings of the National Academy of Sciences* [4-6], including a letter of our own [6], which was signed by over 30 world-renowned experts in phylogenetic analysis, who actually pioneered and contributed to the development of modern phylodynamics. Third, our paper, published in *JMIR Public Health and Surveillance* [7], has also been supported and confirmed by similar findings of other independent investigators [2,8,9], clearly showing that phylogeny-based analyses of SARS-CoV-2 genomic data, available during the early phase of the pandemic, have led to premature conclusions and/or statistically questionable findings, due to a lack of a phylogenetic signal determined by the sudden emergence and exponential growth of the virus, as well as a strong sampling bias. Indeed, our paper [7] shows that even when new (and more recently sampled) sequences are added to the tree, phylogeographic hypotheses of early SARS-CoV-2 spread in Europe, such as the possible introduction of the virus from Germany to Italy, cannot be proven with sufficient statistical robustness, since the sequence data support several other equally likely scenarios.

It is true that methods such as contact tracing and mobile phone tracking can be very effective in tracking outbreaks [10]. Yet, epidemiological tracing and surveillance by other means was not the focus of our work [7], which discusses only the unreliability of using SARS-CoV-2 sequence data, without the aid of other contact tracing methods, to infer virus dissemination during the early phase of the pandemic. Therefore, one of the major points raised by Forster and Forster [1] in their commentary is irrelevant since it is not pertinent to our work or the interpretation of our findings.

Forster and Forster [1] misinterpret the message of our paper, based on targeted sentences/paragraphs taken out of context. There was no trivial or other oversight in our analysis. If anything, the subsequent isolation of sequences of patients from Portugal, Brazil, Wales, and the Netherlands, which were identical to the pre-existing Italian sequence, illustrates our point precisely: “it is not possible with the present data to decide which branching pattern (and, therefore, which phylogeographic reconstruction) most likely represents actual dissemination routes among European countries.” Forster and Forster [1] go on to discuss how the Welsh, both Dutch, and Brazilian patients had all visited Italy a few days before falling ill. This is interesting information that may suggest such patients were infected in Italy; after all, contact tracing is presently considered the golden standard for tracking SARS-CoV-2 dissemination, but it has very little to do with the central problem raised in our paper [7]: branching patterns in the phylogeny alone, especially when based on several identical sequences from different geographic areas (one of the very definitions of a lack of a phylogenetic signal in any basic textbook [11], which Forster and Forster [1] seem to ignore) cannot distinguish among different and equally likely dissemination scenarios. In fact, Table 1 in our manuscript shows, as expected given the presence of several identical sequences sampled over a short time interval in different countries, that alternative topologies underlying alternative dissemination scenarios are equally likely. Besides, the SARS-CoV-2 incubation period and the lack of symptoms during early infection should caution against firm conclusions on the directionality of infection even when further details on travel history are available.

Forster and Forster’s [1] critique of Figure 2 exemplifies the extent of their misreading of our paper [7]. As clearly stated in the legend, the maximum likelihood tree in Figure 2 is displayed as a “cladogram,” which means that branch lengths are not drawn proportional to genetic distance or time-scaled. Cladograms are branching diagrams only showing cladistic relationship among taxa, where branches have an arbitrary length chosen for best display purposes [11]. It seems Forster and Forster [1] misread the figure legend, as the confusion between cladogram and phylogram implied in their rebuttal would be quite egregious for any scientist with a basic background in phylogenetic analysis; hence, they have not discovered any flaw. The fact that previously unsampled sequences from Portugal, Brazil, Wales, and the Netherlands are identical to the Italian sequence is *exactly* the point we are making: such sequences altogether have no phylogenetic signal (defined as the minimum amount of genetic diversity required to generate resolved phylogenies [11]). In a phylogeny with branch lengths drawn proportional to genetic distances, such sequences would appear to cluster tightly along very short branches of actual zero length, simultaneously arising from a common ancestor. This is what we call in the paper a star-like signal, which is obviously associated with phylogenetic noise, that is, the inability to discern the exact evolutionary relationship among sequences (other than to trivially say that they are all identical and, thus, related through a most recent common ancestor).

In summary, while it is true that identical sequences are likely linked by close transmissions, it is also important to remember that, in the absence of phylogenetic information, it would be impossible to establish the correct sequence of events through phylogeny reconstruction alone, which is the whole point of our paper [7].
